# Using photo-elicitation to explore perceptions of physical activity among young people with cystic fibrosis

**DOI:** 10.1186/s12890-019-0985-5

**Published:** 2019-11-26

**Authors:** S. Denford, D. M. Hill, K. A. Mackintosh, M. A. McNarry, A. R. Barker, C. A. Williams, On behalf of Youth Activity Unlimited – A Strategic Research Centre of the UK Cystic Fibrosis Trust, Craig Williams, Alan Barker, Sarah Denford, Eleanor Main, Sarah Rand, Helen Douglas, Mandy Byron, Anne Holland, Narelle Cox, Paul O’Halloran, Kelly Mackintosh, Melita McNarry, Mayara Silviera, Jane Schneiderman, Greg Wells, Jessica Caterini

**Affiliations:** 1 Children’s Health and Exercise Research Centre, Sport and Health Sciences, University of Exeter, St. Luke’s Campus, Heavitree Road, Exeter, EX1 2LU UK; 2 Applied Sports Science, Technology, Exercise and Medicine Research Centre (A-STEM), Swansea University, Bay Campus, Swansea, UK

**Keywords:** Qualitative, Young people, Intrinsic and extrinsic motivation, Social support, Self-determination theory

## Abstract

**Background:**

Physical activity is recommended in the management of cystic fibrosis (CF). The aim of this study was to explore motives, barriers and enablers to physical activity among this population. Methods: Twelve participants (12–18 years) were recruited via convenience sampling. Photo-elicitation alongside semi-structured interviews were used to explore participants’ views and experiences of physical activity.

**Results:**

Our findings revealed motives for physical activity including health, enjoyment and autonomy. Those with families who valued physical activity tended to have positive attitudes towards physical activity, and valued and integrated it into their lives. Moreover, they were likely to be intrinsically motivated to be active. Several factors enable and act as barriers to physical activity. Whilst CF influenced physical activity, the majority of enablers and barriers raised where congruent with the general populations.

**Conclusion:**

This study provides support that healthcare providers should encourage both young people with CF and their families to be active, and subsequently informs the development of clinical interventions to support physical activity among young people with CF and their families.

## Background

Cystic fibrosis (CF) is a life-limiting, inherited condition affecting over 10,000 people in the UK. Treatment of CF is complex and time-consuming [1] and includes a combination of drug therapy, physical therapy and controlled diet. Physical activity, irrespective of whether it is lifestyle or the structured ‘exercise’ sub-component is highly recommended in the management of CF [2], and endorsed by healthcare providers from CF multidisciplinary teams. Exercise, in particular, has the potential to improve important predictors of survival [3], such as exercise capacity and pulmonary function, in both children [4] and adults [5]. Furthermore, physical activity has been shown to have a positive impact on psychological wellbeing, including improvements in health-related quality of life [4, 6–8], an increased sense of mastery over CF [4] and increased sense of “normality” [9]. Despite this, physical activity often declines during adolescence [10], which has lasting implications during adulthood [11].

During adolescence, responsibility for the treatment and self-management of CF shifts from the parent to the individual, whereby the individual dictates whether physical activity is a priority [12]. Even with parental and social support, for physical activity to become an established part of the individual’s life, it has to be something that they are motivated to do [13]. Identification of factors that will motivate young people with CF to be physically active requires a deeper understanding of their physical activity experiences, their life goals and indeed how physical activity can be used to achieve these goals, which may (or may not) be related to the management of CF [14].

Qualitative studies have proposed that, although young people with CF are aware of the importance of physical activity, barriers to being physically active include discomfort [15], worsening of symptoms [15], disinterest [15], lack of time [16], disease burden [16], low self-efficacy [16] and concerns about their physical appearance [9]. Conversely, research also highlights that young people with CF report very few barriers to physical activity [17, 18]. Specifically, young people with CF aged between four and 16 years reported no perceived barriers or restrictions in terms of physical activity, provided their parents acted as enablers [18]. Other studies suggest that parental and clinical support, as well as the presence of strong role models, can act as enablers for physical activity in young people, irrespective of whether they are from a CF population [9] or other clinical and non-clinical populations [19, 20]. However, in contrast to the studies that have explored the health benefits of physical activity [21–24], very little is known about what motivates young people with CF to be physically active (or not), what the key enablers and barriers are, and the role of the family. Exploration of these issues could identify opportunities for healthcare providers to enhance sustainable motivation and support young people to become more physically active throughout their lives [25]. However, in order to obtain insightful and person-centred depth to the raised questions, a qualitative methodology is required.

Photo-elicitation, the inclusion of photographs within a semi-structured interview setting in an attempt to augment the quality of data collected [26], has several advantages over interviews alone. Despite the reliance of researchers on the written or spoken word, it has been suggested that young people feel more confident when using visual approaches [27]. Specifically, photo-elicitation provides an opportunity for young people to be an active participant in the research, to speak for themselves and to ensure their perspective is heard [28, 29]. Moreover, photo-elicitation is recognised as being particularly useful for understanding the experiences of groups, such as young people, because it can overcome the power imbalance between the interviewer and interviewee, thereby decreasing formality, instilling a sense of fun [30] and consequently building rapport [31–33]. Photographs can also evoke a myriad of memories and emotions that can be retrieved and accessed by both participants and researchers during the interview, but also by audiences during dissemination. Photo-elicitation has been used to study the perceptions and experiences of young people [32], people with chronic conditions [18, 34, 35] and in relation to eating behaviors [36], exercise behaviors [37] and physical culture [28, 38]. As such, photo-elicitation will be a useful approach to explore the experiences of physical activity among young people with CF.

The aim of this study is to use photography to explore physical activity from the perspective of young people with CF. Specifically, the study seeks to explore what motivates young people with CF to be active, identify the barriers and enablers to physical activity, and the social and environmental context in which physical activity occurs. Results of this study will enable the development of promotional materials that can be used by healthcare professionals to help young people with CF, their parents and their support team to become more, or remain, physically active, which in turn could lead to significant improvements in both physical and psychological wellbeing.

## Methods

Semi-structured interviews incorporating photo-elicitation were used to explore the views and experiences of physical activity among young people with CF. Participants between the ages of 12 and 18 years with a confirmed diagnosis of CF were recruited via advertisements in CF clinics in the UK, CF social media (Twitter and Facebook) bulletins and via CF networks. Reporting was in line with Standards for Reporting Qualitative Reserach (SRQR; Additional file 2).

Ethical approval was obtained from National Health Service Health Research Authority London Dulwich Research Ethics Committee (17/LO/1321). Participants were asked to capture images (using their smart phone for example) that represented their physical activity behaviors and things that enable or act as barriers to their physical activity levels. Participants were encouraged to take as many photographs as they wanted and to be creative – they could capture images that related to their physical activity either physically or symbolically. Whilst it was not forbidden to take photographs of other people, for ethical reasons, it was stressed that permission must be granted by the individual prior to the image being captured, and that participants should not take photographs of strangers. All photos were emailed to the research team ahead of the interview and included in the analysis.

During the interview, participants were asked to talk about their physical activity and the photos they had taken. They were asked to describe their photos, why they were taken, how it enabled or acted as a barrier to physical activity and how it made them feel (Additional file 1). Questions were informed by existing literature, our own clinical experience and consultations with young people with CF. Whilst it was not possible to obtain an objective assessment of the physical activity levels of the participants, participants were asked whether or not they consider themselves to be active, and asked to describe how much physical activity they typically did each week. Participants who considered themselves to be active, and reported participating in physical activities for at least 30 min at least 5 days a week are described in the analysis as participants who identify as active. In contrast, those who did not consider themselves to be active and reported little to no activity each week are described as participants who identify as inactive. Whilst participants were specifically asked to discuss physical activity, many spontaneously referred to exercise and sport and used the terms interchangeably. Throughout the remainder of this manuscript, we use the term physical activity to encapsulate all movement in the broader sense, irrespective of purpose, intensity and structure.

Interviews were audio recorded, anonymised and transcribed verbatim, and data were analysed using a thematic approach [39]. Two researchers independently read transcripts (and associated photos) and identified core codes. Drawing on existing literature and theory, codes were summarised into a series of related themes [40]. Charts were then developed for each theme [40], with relevant data from each interview cut and pasted into the corresponding chart, which were subsequently used to develop narratives [41].

A variety of techniques were used to maximise the validity of the analysis, including triangulation of the two methods of data collection (photos and interviews), the attention to negative cases and clear exposure of methods of data collection and analysis [42]. In order to ensure sensitivity to context, potential links to existing theories and previous research were noted. A summary of the findings were sent to all participants, along with an invitation to offer any comments (respondent validation).

## Results

A total of 17 young people with CF responded to the advertisements, with 12 participants (seven male, five female) subsequently completing informed assent and parental consent forms. Nine participants described themselves as active (six male, three female), and three participants described themselves as inactive (one male, two female).

The results are presented in three sections: i) reasons/motives for being physically active; ii) factors which enable physical activity behaviors; and iii) factors that act as barriers to physical activity (Table 1). Pseudonyms are presented alongside each quote.

**Table 1:** Summative presentation of three dimensions and respedctive themes and sub-themes for Young people with CF and PA

Dimension	Theme	Sub-theme
Motives	Introjected (extrinsic) motivation	For others [especially parents] Prove people wrong Benevolence / Philanthropy Rewards
	A sense of autonomy	
	Health	General health CF related health / functioning
	Impression management	Look “normal”
	Escapism	
	Positive affect	Enjoyment / pleasure
Enablers	Positive history of PA/Exercise/Sport	
	Social support	Parents [resource / emotional] Peers Relatedness /belonging
	Role Models	Parents CF role model
	Increases in mastery / competence	Including competition
	Positive affect	Enjoyment Fun
	Environmental	Facilities / Technology
	Habituating PA	
Barriers	Health [CF]	Limited time available after treatment Condition limits exercise ability
	Amotivation	Lack of autonomy Lack of competence Comparatively lack of value [for physical activity/ sport]
	Time	
	Negative Affect	Boring [historically] dislike sport
	Environmental / geographical barriers	Weather, facilities

### Motives for being physically active

The participants’ identified several reasons for adopting and maintaining physical activity, including: i) improving/maintaining health; ii) normalising and escapism; iii) gaining autonomy; iv) experiencing positive affect; and v) achieving extrinsic goals.

#### Improving/maintaining health

**Fig. 1:**
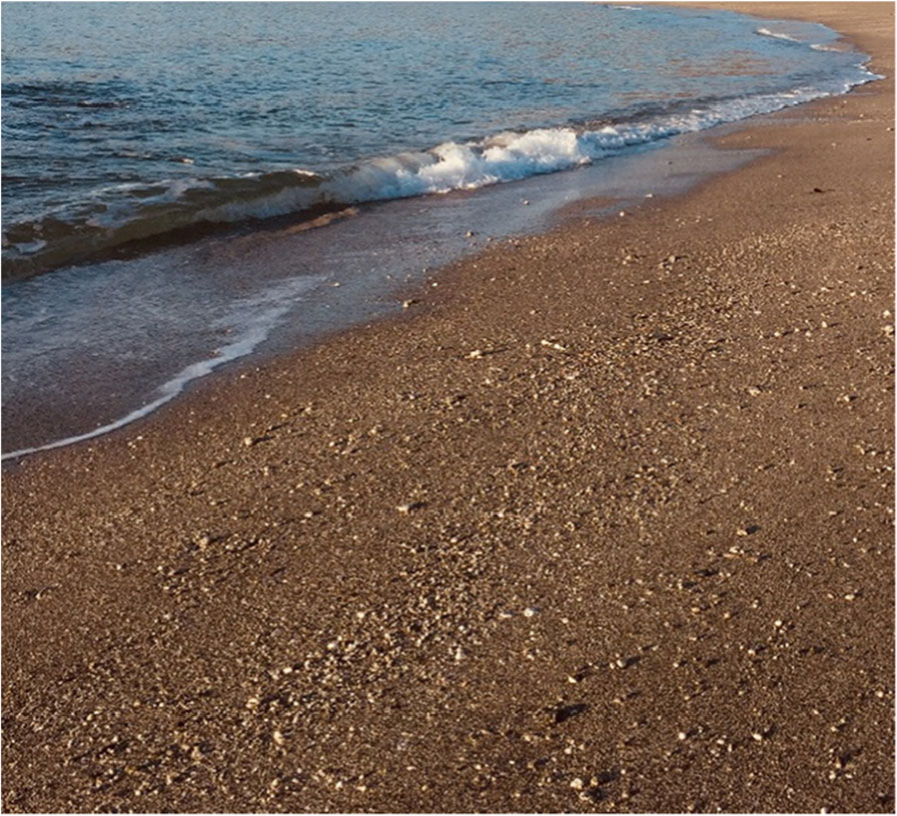
An image of a beach; depicting an opportunity to improve health via swimming. Photos used with permission from study participants

Most participants engaged with physical activity to improve or maintain their health (both specific to CF and general health). In many of these cases, physical activity was adopted to specifically manage their CF condition/symptoms. One participant, speaking about a photo of a beach (Fig. 1), offered the following summary of the highly positive impact that exercise, in particular swimming, was perceived to have on his physical health:

*“I got into exercise and swimming, and I believe that it’s kept me well. For the first 15 years of my life, I was constantly on IVs [intravenous therapy]…My lung function was falling… I was constantly ill…I just thought to myself, ‘I have to do something’…as I couldn’t just sit around and wait for someone to say ‘we’ve found a cure for your CF’…I’ve only been on IVs once since I’ve started [exercising]. I’ve not been unwell for about three years now, other than one time. It’s definitely helped me so much. It’s been amazing. It’s like it’s given me my life back” (Warren, 18 years).*

The positive impact of swimming on lung function was a particularly important motive as it enabled the completion of daily activities:

**“***Yeah [I exercise] just so I don’t get out of breath if I walk up the stairs or up a hill or something” (Claire, 16 years).*

Thus, several participants were evidently driven to push themselves, due to the fear of CF-related ill-health:

*“…I just think like I need to do it to stay healthy. And like, if I want to live a long life, healthy life, I just need to do it and do it properly so I can live” (Henry, 14 years).*

However, it should also be noted that there were a number of participants who were active to improve their general health, rather than for the specific purpose of managing their CF:

*“I think everyone should do more [physical activity], regardless of whether they have cystic fibrosis or not…because it makes you feel so much better” (Jake, 17 years).*

#### Normalising and escapism

**Fig. 2:**
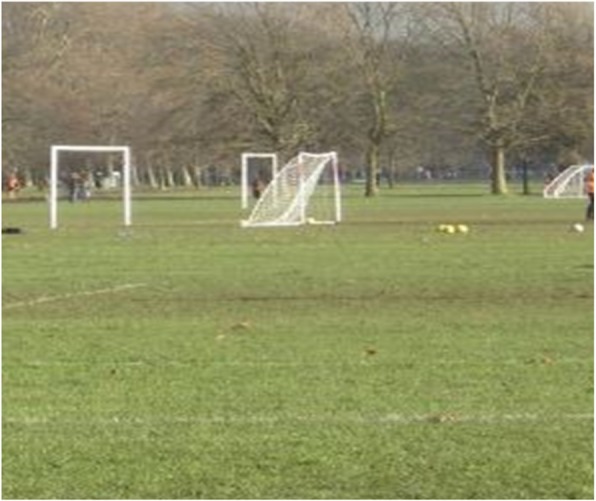
Image of a football pitch; depicting am opportunity for the participant to be “normal.” Photos used with permission from study participants

Interestingly, while physical activity was often undertaken to manage their CF, several participants used exercise/sport to “escape” from CF and experience a sense of normality. As explained by a 17 year old boy in response to a picture of a football pitch (Fig. 2):

*“For me…football was the one thing I did that had absolutely nothing to do with having cystic fibrosis. I mean obviously it did in terms of the implication of cardio on my lungs, but that wasn’t what I thought about. When I was playing football, I was just like everyone else and could forget that I was the kid with that illness…It’s about being normal, and healthy and having fun, and not just being the kid with CF for a while. It’s just about being me” (Jake, 17 years).*

**Fig. 3:**
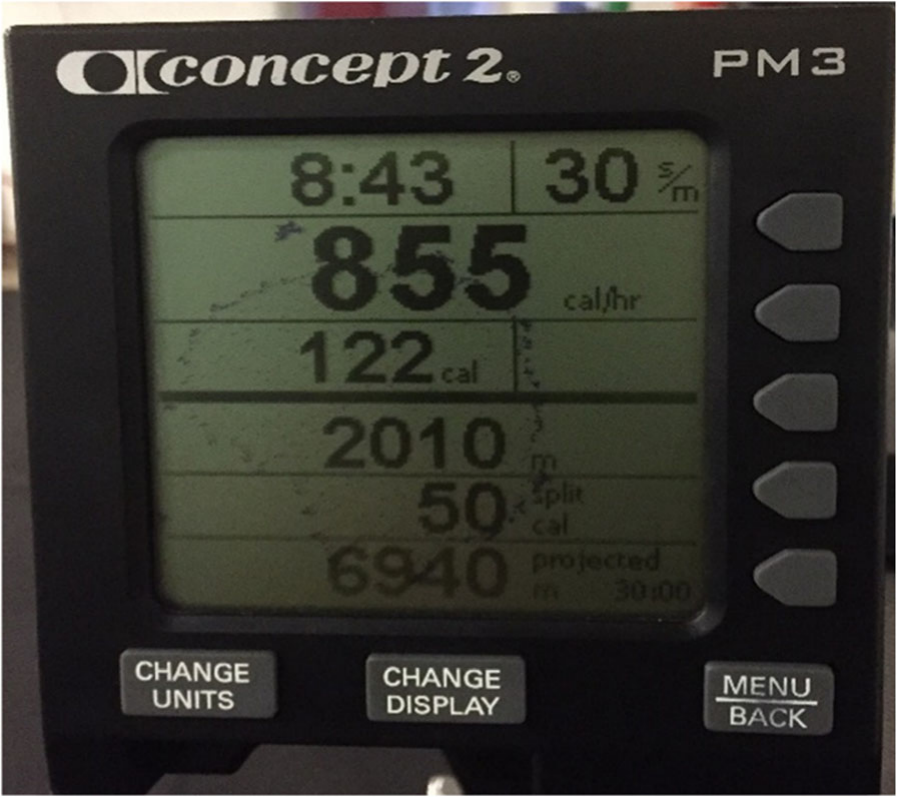
Image of rowing machine; depicting pride in the participants improvement in her ability to row. Photos used with permission from study participants

In addition, a smaller number of participants exercised to escape being ‘seen’ (by others) as, “someone with CF.” Whilst explaining pride for a substantial improvement in her ability to row two kilometres (Fig. 3), one participant claimed:

*“…yeah [I exercise] because I don’t want to be the ill one [laughs]. I used to think that I looked really ill. And I just didn’t want to look like that anymore…I would hate…to have people thinking that because I have CF, I can’t even walk up-stairs” (Claire, 16 years).*

#### Gaining autonomy

It was evident that several participants chose to engage with physical activity because the activity enabled a sense of autonomy. This was considered important for participants, as having CF appeared to reduce the amount of perceived control the participants had within their lives:

*“I really resented having to spend so much time doing things that, in my mind, no one else had to do…My brother, my sister…they could do what they wanted when they got home from school, and I had to do my nebs [nebuliser therapy]…For me, I did it [played football] because it was for me. Not physio, not treatment, not something I had to do. Something that I wanted to do” (Jake, 17 years).*

Such was the desire for autonomy, it was indicated by certain participants that, despite claiming that their main reason for the activity was fun, they would not participate in their chosen sport, if it was prescribed for the ‘treatment’ of CF:

*“I play football because it’s fun…I definitely wouldn’t have been so keen on the idea if I was being told I had to play football” (Jake, 17 years).*

#### Experiencing positive affect

A critical reason to engage with physical activity was for the positive affect the activity brought to the active participants. Thus, they chose to be active because it was “fun” and “enjoyable.” Speaking about a picture of her dog, one participant claimed just looking at the picture made her:

*“Happy. She makes me happy… I love walking her…” (Lucy, 14 years).*

For other participants, the associated enjoyment of the activity enabled them to overcome barriers that appeared to influence the less enjoyable treatment. For example, whilst speaking about the boredom associated with physiotherapy, one participant stated that:

*“Football is different. Football I enjoy, so I can do that whenever”(Henry, 14 years) .*

While many considered physical activity as inherently enjoyable, for other participants, the social support and relational outcomes experienced during the activity, engendered their positive affect. In response to an image of a football team, the participant said:

*“I think as like a team player, like everyone like needs encouragement, because we all encourage each other when we’re playing. And when we encourage, it’s just better… it’s just more fun” (Henry, 14 years).*

#### Achievement of extrinsic goals

All active participants reported they undertook physical activity to achieve a range of extrinsic (external and introjected) motives/goals, including proving other people wrong, and seeking a sense of achievement. For a number of participants, the drive to show others that they were more physically capable than assumed, was a pertinent motive to be physically active. In many of these cases, it was also evident that the frustration associated with others’ low expectations strengthened that motive. When discussing an image of his role model, one participant claimed that:

*“…sometimes at school people think that I can’t do things because I have CF. Like sometimes…the teachers think I can’t do things, and they’re like ‘oh are you allowed to do that? Won’t it make you ill?’…I’m like, ‘no!!, I can still do it’.” (Lucas, 14 years).*

Moreover, and as explained by one participant, CF role models (e.g. Josh Llewellyn-Jones) were an important catalyst for the desire to undertake physical activity to prove others wrong:

*“…like Josh Llewellyn-Jones says, ‘tell me I can’t do something, and I will show you you’re wrong’. And I think that mindset is crucial” (James, 18 years).*

Of interest, this motive also appeared to be associated with the downward social-comparison against those considered to have a more severe form of CF:

*“I did a marathon a few years ago, to raise awareness of CF…For me, my CF isn’t that bad, I have a relatively mild mutation. So that is one of the other things that motivates me. Because I can do it, and I think that [there are] so many people with CF who would like to do more activity, but can’t. So…I make myself run. And that’s why I did the marathon.” (Matt, 18 years).*

Those participants who engaged with physical activity to pursue a sense of achievement, valued the competitive nature of exercise and sport:

*“Having someone there who maybe can lift more, or who can get a better time… it’s like having something to aim for, and it keeps you going just that little bit more” (Claire, 16 years).*

Indeed, striving to achieve an exercise goal was considered by some participants, to be more important due to their CF:

*“…you know it’s [exercise] going to be hard…you know it’s going to be painful…But that sense of achievement at the end…And that’s where having CF gives me an edge… I’m used to pushing through. Where maybe some of the others are a bit quicker to stop. But then, for them, the stakes aren’t quite as high” (James, 18 years).*

### Enablers of physical activity behaviors

The key factors perceived by the participants to encourage/support the adoption and maintenance of physical activity were: i) social support; ii) a positive history of physical activity; iii) role models; v) physical mastery/competence; and vi) positive affect.

#### Social support

All active participants identified that their physical activity was enabled through social support provided by their family. In each case, the family had offered tangible support in terms of accessing physical activity experiences. As an example, in response to an image of her mother, a 15 year old girl explained:

*“She [their mother] drives me to every dance class I want to go to, she waits for me while I’m there, she comes to every single performance. She’s the best. And she never makes me do my homework before I go because she knows how important my dancing is. She’s like ‘oh you’ve been sat down all day, I’ll speak to your teacher’” (Abby, 15 years).*

**Fig. 4:**
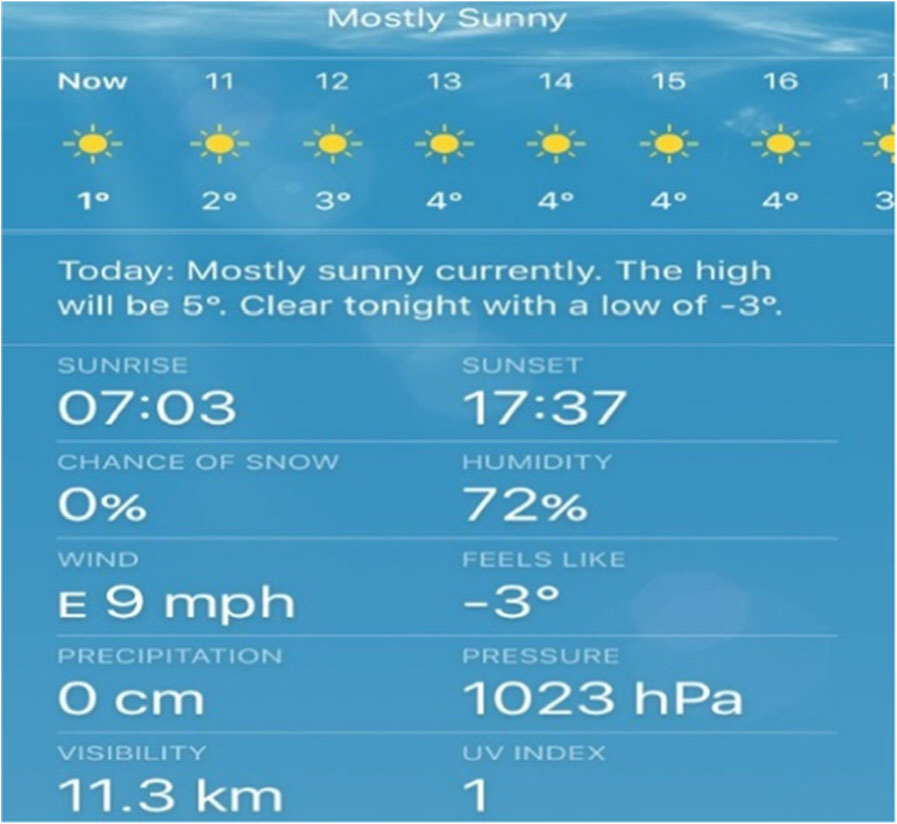
Image depicting a potential barrier to physical activity that requires additional support to overcome. Photos used with permission from study participants

Moreover, the family unit often offered emotional and motivational support, which was critical to sustaining the participants’ physical activity. In response to an image representing bad weather (Fig. 4), one participant highlighted his need for social support and encouragement to enable them to overcome the barrier:

*“Yeah …my mum…it’s like if I couldn’t be bothered to go [running] she would encourage me, and I would feel like I was letting her down if I didn’t go” (Matt, 18 years).*

As the participants moved to/through adolescence, the influential source of social support seemingly moved from the family towards their peers. Therefore, having friends who were supportive of physical activity and contributed positively to their physical activity experience, was a critical factor in their maintained activity. When asked about an image of the participant with a friend, and the role that the friend played in supporting and encouraging physical activity behavior, the participant explained:

*“Just by making the classes more fun…Yeah she makes it more fun.. And she makes me go to the fitness classes…well she doesn’t make me!!… but she’s always like ‘oh come on…, come with me’. If I’m like ‘I’m too tired’ or’ too much homework ‘or anything… I try and back out (laughs), but [friend] doesn’t let me.” (Abby, 15 years).*

In addition, a small number of participants identified that the social support offered by their CF medical team enabled their physical activity behavior. As an example:

*“…[the] physios at the hospital…are really great and encouraging, and get you up and about even when you just want to lie there and pity yourself… And that really does help your mindset” (James, 18 years).*

#### Positive physical activity history

For those participants who had maintained their physical activity over an extended period of time, it was evident they had been exposed to constructive physical activity experiences throughout their childhood (via their family unit). This in turn, had encouraged their positive attitude towards physical activity, and the integration of physical activity into their lives. As explained by one participant:

*“…when I was little, my parents were really dedicated runners, and I think that growing up with fitness is really important. Just to have that upbringing and…be exposed to fitness. I guess that’s had a really key role in making me who I am today…so I have always been fairly active” (James, 18 years).*

Similarly, another participant noted:

*“There was a big emphasis during my upbringing…the way parents dealt with physio and exercise from day one…Like if I miss one session it wouldn’t kill me, but it’s like a religious practice. The Muslim has to pray 5 times a day. [Exercise and physiotherapy] is just what you do, and if you didn’t do it, you would feel bad about it” (Matt, 18 years).*

#### Role models

It was proposed by several participants that prominent individuals with CF, who had reached very high levels of fitness (including professional sport), had impacted their physical activity behavior significantly:

*“…like Josh Llewellyn-Jones… very fit inspirational guys…they’re an inspiration for ‘what can CF patients do’. And I think if you’ve got a kid that’s just been diagnosed…there is so much about the negatives and what you can’t do. And it’s good to have the opposite message of ‘don’t be despondent - look what you can do if you set your mind to it’. It’s what I tried to do with the marathon” (Matt, 18 years).*

Similarly, another participant noted that:

*“Ben Mudge….I just think he’s really inspirational and he makes me want to be more active too…I think he’s just like really fit and active and um, because he has CF…and he does loads of fitness stuff…it just makes me want to do more active stuff too*” (Lucas, 14 years).

It appeared that the role models were able to enhance the participants’ self-efficacy towards physical activity, and, as a consequence, their motivation to become active:

*“I think it’s always just good to see others…in similar situations as yourself, really doing something…and doing it well. He’s [Josh Llewellyn-Jones] really successful…that just gives people a bit of hope and a bit of encouragement when they need it. You know, just a reminder that you don’t need to let CF stop you having a life, that you can still do everything you want to do” (James, 18 years).*

#### Mastery/competence

In order to enable the maintenance of physical activity, the active participants identified the importance of perceived competence. This was particularly the case for participants discussing the exercise and sporting aspects of physical activity:

*“…back then I was a lot less fit, and I mean a lot less fit, and it was scary [to exercise]. It’s really scary taking that first step, not knowing if you’re going to be able to make an improvement. But…you do eventually get better and better…It’s a long way from where I started. And I think it’s important to remember how far you’ve come” (Warren, 18 years).*

Critically, significant others (e.g., parents, peers and the supporting medical team) were able to positively influence the participants’ perceived competence within the physical activity environment. As an example, one participant recalled that:

*“I didn’t want to go [to the gym] by myself, so my physio at the hospital came with me a few times and then I started personal training” (Claire, 16 years).*

#### Positive affect

Participants who remained active over a prolonged period identified that their chosen physical activity elicited positive affect. Indeed, the consistent message provided was that they maintained their physical activity due to the activity being either innately “enjoyable” or through significant others (friends, family, medical support team), making the experience “fun”.

### Perceived barriers to physical activity

The main perceived barriers to physical activity reported by the participants included: i) health; ii) time; iii) value of physical activity; iv) negative experiences of sport and exercise; and v) environmental.

#### Health

The participants who were less active/sedentary, noted that their CF-related ill health often acted as a barrier to their physical activity. As summarized by one participant:

*“…it such a vicious circle…you want to be active, you want to get fit, but as soon as you start to get into it, you get ill and lose all the fitness and you’re right back to square one” (James, 18 years).*

Of note, a smaller number of participants also recognised that episodes of poor mental health that had been actuated by their CF, also limited their willingness and perceived ability to exercise. Speaking about an image of his younger self, one participant stated:

*“I was so poorly …I had no body fat, no energy, in and out of hospital…no motivation, just feeling sorry for myself the whole time….I would say that I was depressed, and that I did suffer from depression for a while…I just didn’t care about anything to do with CF…I just wanted to be left alone…I didn’t believe that I could do anything…I just thought it was unfair” (Warren, 18 years).*

#### Time

**Fig. 5:**
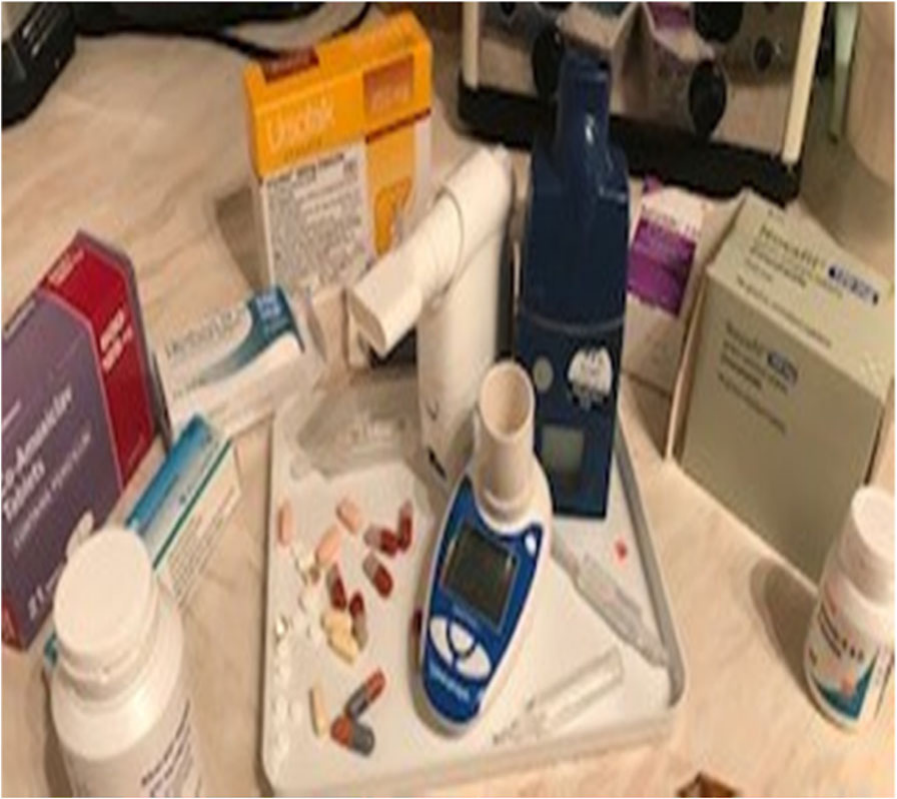
Image depicting a stringent medication regime. Photos used with permission from study participants

Certain participants identified that the time demands associated with managing their CF was a key barrier to exercise. Speaking about an image of medication (Fig. 5), one participant claimed:

*“So…my nebuliser and my tablets, and I do my physio in the evening too. All together it takes an hour, or maybe two. And I don’t really want to do anything else in the evening after all that” (Isabelle, 13 years).*

Moreover, other activities/tasks such as schoolwork and part-time work were proposed to leave little time for physical activity. In response to an image of her homework, one participant noted:

*“…when I have too much homework…it stops me being active …we get loads” (Lucy, 14 years).*

While another stated:

*“Yeah, I have to spend all my spare time revising, and then don’t have any time for sport” (Heidi, 16 years).*

#### Value of physical activity

While time was perceived as a hindrance to physical activity for some participants, this appeared to be tempered by the individuals valuing physical activity less than other tasks. That is, they chose to participate in other activities during their (limited) leisure-time that was considered to be more enjoyable. As elucidated by one participant whilst speaking about an image of her place of work;

*“…I have to work all day, well most of the day on Saturday, and then I only really get one day off a week and I don’t want to waste it doing something I don’t want to do” (Heidi, 16 years).*

Likewise, in response to an image of a shopping centre, she stated:

*“I would just rather do that [shopping with friends] than exercise on my day off. Um, yeah, I would just rather be with my friends” (Heidi, 16 years).*

While another participant explained how their “boring” CF treatment drove them to specifically seek activities that were more enjoyable than physical activity. In response to an image of her medication one participant stated:

*“It’s just that I think because it’s [physiotherapy] quite boring having to do it every day, I don’t really want to have to do something else [e.g., physical activity] boring as well” (Isabelle, 13 years).*

#### Negative experiences of sport and exercise

Many of the inactive participants had developed a dislike of physical activity through negative past experiences. Interestingly, negative experiences appeared to specifically relate? to sport and exercise, rather than physical activity. For the most part, those negative experiences had lowered their level of perceived physical competence/self-efficacy. As an example:

*“The swimming pool used to be split into lanes, and so you had to swim up and down the lanes. And I couldn’t keep up. I would have people overtaking me and kicking me, and have their feet and water in my face. It really put me off… and I stopped going” (Matt, 18 years).*

While another inactive participant described how anxious the image of her sports hall made her feel, and acknowledged the reason they avoid taking part in exercise or sport:

*“I‘m not very good at PE…I can‘t ever do anything and it‘s just really really embarrassing. Whenever we do anything, I come last…We were playing rounders the other day and I was the only person who couldn‘t hit the ball. The only person!!. It was so embarrassing” (Isabelle, 13 years).*

#### Environmental

**Fig. 6:**
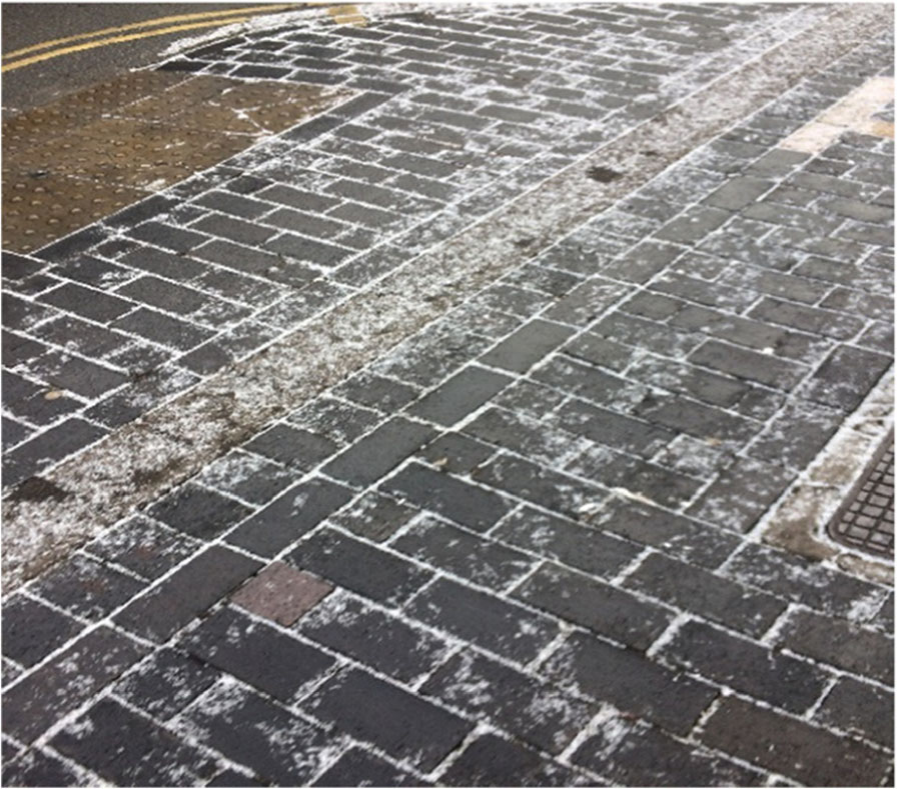
Image of bad weather preventing physical activity. Photos used with permission from study participants

Finally, weather conditions and safety concerns were proposed as barriers to physical activity. Whilst discussing an image of predicted cold weather (Fig. 6); one participant stated:

*“When it’s cold and raining…I don’t want to go out and run then…it’s like a battle. And I’m really restricted with when I can run, because…I don’t like the idea of running around [home city] in the dark, especially in the centre because that could attract strange people and I don’t feel safe” (Claire, 16 years).*

## Discussion

Our aim was to explore physical activity from the perspective of the young person with CF using a novel approach of photography. To the best of our knowledge, this is the first use of this photography technique, in combination with semi structured interviews in CF research, to identify the complex interplay of barriers and enablers to physical activity. Understanding of these issues is imperative if healthcare providers are to support young people with CF to be physically active. Overall, it is evident that there are several barriers and enablers to physical activity within young people with CF. Whilst some were explicitly related to CF, many factors, although clearly influenced by having CF, appeared to be a reflection of the participants’ generic relationship with physical activity. Critically, motives for physical activity varied considerably between individuals, thus highlighting the need for an individualised approach to physical activity promotion. Whilst this may appear inherently obvious, many interventions targeting physical activity still adopt a “one size fits all” approach, such as giving patients fitness trackers [43] or educating individuals about the benefits of physical activity [44]. For interventions to be effective for enhancing physical activity, our data/study suggests that they must be flexible enough to meet the various motives of those they target. Within the current research, motives for physical activity could be considered physiological, psychological and social, each of which should be considered in the development of future interventions [45].

Initiatives such as “Exercise is Medicine” [46] recognise the importance of physical activity in preventing, managing and treating a range of chronic conditions. Advocates of such initiatives call upon healthcare providers to prescribe physical activity in the same way that they would prescribe a drug treatment. Indeed, many of our participants agreed that physical activity could be used to prevent declining health (and even delay death), improve poor health (e.g., sustained periods without illness) and for symptom management (e.g., improved abilities to perform every-day activities). This finding emphasises the need for clinical teams to focus not just on the importance of physical activity for improving clinical markers (e.g., lung function), but to highlight the impact it may have on outcomes that are important to patients (e.g., sustained periods without illness) and have a direct influence on individuals’ day-to-day lives, as these outcomes are more likely to be motivational than outcomes prioritised by clinical teams. However, participants also recognised that physical activity should not *just* be medicine. Many of the participants were motivated by the generic health and fitness benefits of physical activity and noted that everyone should do physical activity, not just those with CF. Furthermore, prescribing physical activity as a drug may have the undesired effect of making physical activity something that the patients feel they *have* to do, rather than something they *want* or *choose* to do and is therefore less likely to be sustainable. Once individuals start to feel pressure to be active, irrespective of whether it’s internal or external, motivation for physical activity becomes “controlled” rather than autonomous [13], and physical activity could become less enjoyable and start to decline [13]. Indeed, within the current study some participants explicitly state that they would not have been active if they had been told that it was something that they *had* to do. This finding suggests that for some, promoting ‘exercise as medicine’ could be detrimental for sustainable, positive, or enjoyable physical activity, and could actually contribute to a decline in physical activity. This finding critically highlights the need to explore patients’ motivations for physical activity before promoting physical activity as a treatment.

The findings in this study also highlighted a complex interplay between barriers, enablers and motives for physical activity, with “social support” being a central theme. Many participants spoke of tangible and emotional support they received from their parents. Indeed, tangible support enabled young people with CF to overcome some of the structural barriers to physical activity (e.g., time, environment) by intervening on behalf of the young people with CF, such as speaking to teachers or providing transport to activities. However, families were also able to promote physical activity through provision of a positive experience of physical activity throughout childhood, increasing positive affect by engaging in activities with the young people with CF and offering encouragement and support to increase mastery and competence of physical activity. Families could also instil positive values for physical activity among young people with CF, thus overcoming barriers related to low value for physical activity. This finding aligns with previous research into the role of the family in enabling physical activity among young people with CF [18] and other populations [47, 48], and strongly highlights the need to incorporate families and/or friends in future interventions [49].

The role of social comparison also appeared to be influential, particularly for those motivated by the desire to demonstrate capability – both to others and themselves. For these participants, the presence of others who are either *more* or *less* able than themselves, increased motivation. Social comparison theories differentiate between upward (i.e., comparing to individuals performing better than themselves) and downward social comparison (i.e., comparing to individuals performing less well than themselves). Upward and downward social comparison can be motivational (e.g., by boosting an individuals’ perception of themselves relative to another, or by providing a role model) or harmful depending on individual personalities [50]. Whilst it is important to note that social comparison can be detrimental, social comparison appeared to increase motivation within the current study. Furthermore, role models were a key enabler to physical activity participation, both in terms of inspiring people to be active and emphasising the message that you can be active with CF. This strategy suggests that the inclusion of a social comparison element in interventions could potentially be effective for some, but not all. However, due to the potential for detrimental comparisons, such interventions must be used with caution.

Motives identified through this research are compatible with the self-determination theory, which underpins many interventions targeting motivation for physical activity [51]. According to the self-determination theory [13], motivation can be intrinsic or extrinsic, internal or controlled. At one end of the spectrum is intrinsic motivation, referring to motives such as enjoyment, interest and autonomy. At the other end of the spectrum is externally regulated motives, such as seeking external rewards or the avoidance of punishment. According to the theory, motives that are internal are more likely to lead to sustained behavior than those that are externally driven. Within the current research, motives for physical activity include autonomy and enjoyment but also health promotion. For these individuals, outcomes of physical activity (i.e., health, fitness) had significant personal importance (identified regulation) and physical activity was therefore maintained.

The self-determination theory suggests that those who are “amotivated” either do not value the activity (e.g., do not experience pleasure from being active) or the outcome of the activity (e.g., do not value the potential impact on health), or they do not feel competent in their ability to perform the activity. Indeed, within the current research a key barrier to being active was not valuing physical activity or its outcome. Furthermore, low value for physical activity also appeared to influence participants’ ability to overcome some of the other potentially modifiable barriers; time, health, and access to facilities also appeared to be a barrier to physical activity participation.

Interventions compatible with the self-determination theory include techniques that focus on building autonomy through facilitating the individuals’ point of view, acknowledging the patients’ perspectives, and building competence for the activity [51]. Theories such as motivational interviewing, which is a collaborative, person-centered approach to support change in a way that is compatible with the values of the individual [14], are also highly compatible with the current research. Future research involving the development of interventions for young people with CF may benefit from the integration of self-determination theory and motivational interviewing for facilitating change in a manner that is tailored to the needs and values of the individual.

### Implications for policy and practice

This research has a number of implications for policy and practice. First, this research highlights the need for healthcare providers to explore the motives (e.g., using motivational interviewing) of each individual patient, which may not align with the priorities of the healthcare provider. Indeed, attempts to “sell” physical activity to young people with CF are more likely to be effective if their individual priorities have been established [52]. This would not only allow the healthcare provider to highlight the role of physical activity in achieving the young person with CF’s goals but would also allow them to provide truly tailored advice regarding the construct (type, frequency, intensity, duration) of physical activity that would enable the young person with CF to achieve any set goals. Previous work has highlighted considerable variation in the recommendations provided to young people with CF [53]. However, the need for advice to be “individualized” or tailored to the individual circumstances of the young person with CF was strongly emphasized [53]. However, the implementation of ‘individualized’ physical activity appears to be some way off as a greater understanding of which factors need to be taken into consideration is required. The current research offers further support for the need to explore factors beyond CF and its management; for example, the current research highlights the need for healthcare providers to identify the importance placed on a range of motives for physical activity (e.g., health, enjoyment), as well as the need to identify the importance placed on physical activity by the individual’s families and friends. Interventions such as motivational interviewing may support healthcare providers better understand these issues. This will ensure that advice relating to physical activity is truly person centered and individualized.

The role of the family and friendship groups appears to be integral to physical activity and impacts on both motives for physical activity, and sustaining physical activity in the long-term. This inclusion highlights the urgent need to promote families in interventions to support young people with CF to be more physically active. Despite this, and other research [18], highlighting the role of the family in promoting physical activity [48], families are rarely the target of interventions promoting physical activity for young people with CF.

### Strengths and limitations of this work

A key strength of the present research is the use of photography to elicit detailed accounts of the views and experiences of physical activity among young people with CF. The inclusion of photo-elicitation in interviews provided participants with visual reminders of their physical activity experiences, and offered the researcher an opportunity to view physical activity through the lens of the participant. Asking young people to describe how they felt looking at the images provided insight into the every-day experiences of the participants. By giving participants the opportunity to take pictures of their choice, we were able to capture a range of experiences as lived by the individual. Multiple strategies were used to enhance the validity or trustworthiness of the data, including multiple experienced coders and respondent validation [39]. Our findings were interpreted with reference to existing literature, and we asked participants to comment on the relevance of our findings.

A limitation of the current study is that our sample is possibly biased towards those with a personal interest in physical activity. However, despite many of the participants reporting being active on a regular basis, this was not the case and some participants reported that they were not very active at all. This range of perspectives allowed us to explore motives, enablers, and barriers to physical activity among those who identify as active, and those who identify as inactive. This is critical if we are to develop interventions to support young people with CF to be more active.

## Conclusion

This is the first study using photo-elicitation to explore physical activity motives, barriers and enablers among a group of young people with CF. Critically, the analysis revealed a range of motives for physical activity from which clinical interventions to enhance motivations for physical activity can be developed. Whilst several factors that enable and act as barriers to physical activity were identified, many factors appeared to be a reflection of the participants’ relationship with physical activity, which had been influenced by their CF. The present findings show that those with families who are supportive of physical activity tended to have a positive attitude toward physical activity, valued and had integrated physical activity into their lives, and were more likely to be intrinsically motivated to be active. Further research is needed to develop interventions to increase motivation among less motivated individuals, in which healthcare professionals work with young people and their parents to identify individual goals related to activities and lifestyles, and promote involvement in a range of activities in order to identify those that spark enjoyment among the young person.

## Supplementary information

**Additional file 1.** Semi structured interview questions. Questions used to structure the semi structured interviews with participants

**Additional file 2.** SRQR checklist. Completed SRQR. Details pertaining to standards for reporting qualitative research

## Data Availability

All data generated or analysed during this study are included in this published article.

## Abbreviations

CF: Cystic fibrosis
IV: Intravenous therapy
