# Supplementary material for: Using photo-elicitation to explore perceptions of physical activity among young people with cystic fibrosis
Source: BMC Pulm Med. 2019 Nov 26;19:220. doi: 10.1186/s12890-019-0985-5 (PMC6878712; doi:10.1186/s12890-019-0985-5)
Supplement: Supplementary file 1 — Additional file 1. Semi structured interview questions. Questions used to structure the semi structured interviews with participants [file 12890_2019_985_MOESM1_ESM.docx]

Additional file 1: Semistructured interview questions

1. Can you tell me a bit about your physical activity – what sort of physical activities do you do?
   1. How often do you do physical activity?
   2. Do you enjoy being active?
   3. Where, when and with you do you usually do your physical activity?
   4. What are your main reasons for doing your physical activity?
2. How many photos have you chosen to talk about? Which would you like to start with?
3. Tell me about this photo
   1. What’s happening in this picture?
   2. Why did you take it?
4. How were you feeling when you took this picture?
   1. Why?
5. Is the picture of something that stops you being physically active, or helps you to be physically active?
   1. Why is this?
   2. How often does the image help / prevent (as appropriate) you being active?
6. (If prevents) What sort of things could we do to help you overcome this problem and be more physically active?
7. Repeat question 3-5 for each photo
8. Do you think we’ve talked about the main things that help you to be physically active, and the things that stop you being active? Is there anything else that we haven’t talked about yet?
9. Is there anything else you want to tell me about your physical activity?
